# Religious Coping Strategies for Religious Patients with Schizophrenia and Schizoaffective Disorder

**DOI:** 10.1192/j.eurpsy.2022.633

**Published:** 2022-09-01

**Authors:** E. Gedevani, G. Kopeiko, O. Borisova, T. Vladimirova, V. Kaleda

**Affiliations:** FSBSI Mental Health Research Center, Researching Group Of Specific Forms Of Mental Disorders, Moscow, Russian Federation

**Keywords:** Religious Coping, remission in schizophrenia, schizophrénia, Schizoaffective disorder

## Abstract

**Introduction:**

Over last decades convincing evident data was accumulated about the positive correlation of religious involvement and better mental health in depression, substance abuse, suicide, stress-related disorders and dementia. The studies of the impact of religion on patients with schizophrenia and schizoaffective disorders are still insufficient and controversial.

**Objectives:**

To investigate the impact of the religious coping strategies in patients with schizophrenia and shizoaffective disorder with focus on resolution and quality of remission in schizophrenia and schizoaffective disorder.

**Methods:**

The pilot 1 year study covers 68 orthodox (group 1) and 55 unbeliever (group 2) outpatients with schizophrenia and schizoaffective disorder in remission on the maintenance therapy in FSBSI MHRC. The groups matched in age (18-60 y.o.), gender, treatment. The orthodox group received religious coping therapy. Number of relapses, remission quality (PANSS), quality of life (QLS), compliance (MARS) were measured 3 times (baseline visit, 6, 12 months). Statistical analysis (regression and correlation) was applied.

**Results:**

Religious coping strategies proposed by Paragment K. (2013) were applied considering the peculiarities of value-semantic structures and selected religious values of the patients as important rehabilitation resource (Kopeyko et al, 2016). Group 1 demonstrated statistically significant better remission at 12-months point – better subjective well-being, social/functional outcomes, higher adherence to medication, less relapses, less psychotic symptoms.

**Conclusions:**

Religion contributes to acquire adaptive functions as the meaning of life, sense of hope, spiritual comfort, supports the overcome the disease burden. Religious coping is an important tool for rehabilitation and preventing the relapses in patients with schizophrenic disorders.

**Disclosure:**

No significant relationships.

